# Stent-Assisted Angioplasty (SAA) at the Level of the Common Femoral Artery Bifurcation: Long-Term Outcomes

**DOI:** 10.1007/s00270-020-02413-9

**Published:** 2020-01-23

**Authors:** H. Stricker, L. Spinedi, C. Limoni, L. Giovannacci

**Affiliations:** 1 Department of Vascular Medicine, Ospedale La Carità, Locarno, Switzerland; 2 University of Applied Sciences and Arts of Southern Switzerland, Lugano, Switzerland; 3 Department of Vascular Surgery, Ospedale Civico, Lugano, Switzerland; 4 Department of Angiology, Ospedale La Carità, via all’Ospedale 1, 6600 Locarno, Switzerland

**Keywords:** Common femoral artery, Stenting, Peripheral artery disease, Long-term follow-up

## Abstract

**Background:**

The objective of this retrospective single-center study was to report the initial and the long-term outcome after stent-assisted angioplasty of occlusive disease at the common femoral artery.

**Materials and Methods:**

Between 1995 and 2015, 94 limbs in 79 consecutive patients (54 men; mean age 70 ± 8.6 years) underwent angioplasty with self-expanding stent implantation in 94 common femoral arteries. Critical limb ischemia was present in 15 limbs (16%); the other patients had claudication.

**Results:**

Technical success was 99%. Complications occurred in 5/94 interventions (5.3%): puncture site hematomas (2), arteriovenous fistula (1), cholesterol embolism (1), and dissection of the access site artery (1). The intervention was outpatient-based in 98%. Median follow-up was 53 months. Ankle–brachial index (ABI) rose from 0.71 ± 0.17 to 1.0 ± 0.2 (*p* < .001) immediately after the intervention and was 1.03 ± 0.2 after 1 year and 0.96 ± 0.21 at the last follow-up visit (*p* < .001 compared to pre-interventional ABI). During follow-up, restenosis was found in 23/94 limbs (25%); 15 limbs were treated by angioplasty, 3 by surgery, and 5 conservatively. One limb was amputated below the knee 6 months after stent-assisted angioplasty (SAA). Death rate during follow-up was 35/79 patients (44%).

**Conclusions:**

SAA of the CFA resulted in high immediate success and a low complication rate. Restenosis rate was moderate, and target lesions could easily be retreated by angioplasty. The main hazard was not restenosis, but death during follow-up.

## Introduction

Common femoral artery (CFA) disease is felt to be a territory where the surgical approach is superior to angioplasty. The most recent guidelines of the European Society of Cardiology (ESC) recommend surgery over an endovascular strategy, and stenting is not considered for bending areas such as hip and knee joints as well as in arterial segments which may serve as a landing zone for potential bypass, which includes the CFA [1]. Actually, open repair of CFA lesions has shown excellent primary patency [2–4] and long-term results with limb salvage rate up to 100% after 7 years [5]. However, common femoral endarterectomy (CFE) has its downsides including wound infections and lymphatic complications, which may result in a prolonged hospital stay. In a French study, CFE resulted in a local morbidity rate of 21.6%, a need for re-interventions of 3.6%, and a perioperative mortality of 1% [6], while others found major complications in 5% resulting in re-intervention and minor complications in 9% requiring prolongation of hospital stay [3].

Endovascular treatment of CFA lesions is appealing due to its less invasive nature as compared with CFE, the possibility of outpatient treatment, and the avoidance of infection and lymphatic complications. Over the last years, there have been a growing number of endovascular treatment studies published, which were mostly retrospective, frequently including a relatively small number of patients, reporting limited follow-up time, and using different techniques, in particular with respect to stenting [7–11].

With this in mind, we present our retrospective, monocentric study with a long-term follow-up of a consistent number of patients with CFA lesions treated by stent-assisted angioplasty (SAA) according to an established treatment protocol.

## Materials and Methods

### Patients

A retrospective analysis of all patients treated by SAA of the common femoral artery was done using prospectively collected standardized follow-up data between 1995 and 2015 at our institution. We chose an endovascular-first therapy in consecutive patients with common femoral artery obstruction irrespective of their eligibility for surgical endarterectomy. All patients were treated with endovascular therapy with the exception of those who had an impediment for the procedure, i.e., the impossibility of a crossover approach in case of previous aorto-iliac bypass surgery or a contralateral iliac occlusion. Accordingly, 19 limbs had to be treated surgically in the same time frame. The results of a few patients included in this series have previously been published [9].

The obstructions at the CFA included lesions located at the external iliac artery and extended to the CFA, lesions limited to the CFA, and lesions located at the CFA and its bifurcation. We excluded patients with anastomotic stenosis after previous bypass surgery. Typically, the lesions consisted in eccentric calcified plaques; in 10 limbs the CFA was occluded. The runoff was compromised in 26 limbs with occlusion of the superficial femoral artery (SFA). Informed consent for the procedure was obtained from all patients.

The patients were classified according to the Rutherford classification from stage 2–5 with CFA de novo lesions and a diameter reduction of the vessel of more than 50% (determined by a peak systolic velocity ratio > 2.4) at duplex sonography, computed tomography scan (CT), or magnetic resonance (MR).

In all patients measurement of the ankle–brachial index (ABI) was performed; they had either claudication (Rutherford 2–3) or critical limb ischemia (CLI; Rutherford 4–5). Assessed risk factors included patient age and gender, hyperlipidemia, diabetes mellitus, arterial hypertension, body mass index, coronary heart disease, end stage renal insufficiency, cerebrovascular disease, and cigarette smoking (Table 1).

**Table 1:** Demographic, baseline treatment, and risk factor data of 79 consecutive patients and 94 limbs undergoing stent-assisted angioplasty of the common femoral artery

Age, years (± SD)	70 (8.6)
Men, *n* (%)	54 (68)
Arterial hypertension, *n* (%)	68 (86)
Diabetes mellitus, *n* (%)	37 (46)
Dyslipidemia, *n* (%)	45 (57)
Body mass index (± SD)	25.6 (4.8)
Smoking history, *n* (%)	62 (78)
ESRD, *n* (%)	3 (4)
Coronary heart disease, *n* (%)	30 (38)
Cerebrovascular disease, *n* (%)	18 (23)
Critical limb ischemia, *n* (%)	15 (16)
Aspirin or clopidogrel, *n* (%)	79 (100)
Statins, *n* (%)	64 (81)

### Procedure

Patients were treated by either aspirin (100 mg/day) or clopidogrel (75 mg/day) for at least 1 day prior to the intervention. On the day of stenting procedure, double antiplatelet therapy was initiated (with 300 mg of clopidogrel or 500 mg of aspirin, respectively) to be continued for 1 month (with clopidogrel 75 mg or aspirin 100 mg qd, respectively), with single drug antiaggregation thereafter. Routinely prescribed statins were tolerated in 81% of the patients (Table 1). Angioplasty and stenting were performed by contralateral approach, using a dedicated crossover 6 or 7F sheath. Heparin (5000 U) was administered intra-arterially through the sheath. After successful guidewire passage of the target lesion, predilatation was performed with a 1-mm undersized balloon with respect to the vessel diameter. A variety of exclusively self-expandable stents were used to cover the lesion: Absolute Pro Vascular (Abbott Vascular, Santa Clara, CA, USA): 48%; Zilver Flex Vascular Stent (Cook Europe, Bjaeverskov, Denmark): 13%; Wallstent (Boston Scientific, Marlborough, MA, USA): 12% (stainless steel); Sentinol (Boston Scientific, Marlborough, MA, USA): 9%; Epic (Boston Scientific, Marlborough, MA, USA): 8%; Jostent (Jomed GmbH, Rangendingen, Germany): 7%; Everflex (Medtronic, Galway, Ireland): 2%; and Xceed (Abbott Vascular, Santa Clara, CA, USA): 1%. Final dilatation was done with balloons that matched the size of the stent. In case of occlusion of the superficial femoral artery, the stent was placed from the CFA into the deep femoral artery. The length of the stents was between 4 and 6 cm in 83 limbs and between 80 and 90 mm in 11 cases, and the size of the stent was chosen to match the vessel’s diameter plus 1 mm. The origin of the deep femoral artery was left untouched whenever possible or dilated if stenosed; in case of plaques that extended into the superficial femoral artery (SFA), the deep femoral artery (DFA) was “jailed” without impeding the flow into that vessel. We used closure devices (StarClose, Abbott Vascular, Santa Clara, CA, USA) in 80%. The interventions were considered successful if there was < 30% residual stenosis on the angiography acquired in two planes.

Control of success was performed the same day and included clinical assessment and ABI measurement; in case of insufficient improvement of the ABI, duplex sonography was performed. Successful intervention was defined as an increase in ABI of at least 0.15 or a peak systolic ratio at duplex scan < 2.4 across the dilated area. Patients were treated on an outpatient basis if no complication such as major hematoma or false aneurysm occurred at the puncture site, and if there was no sign of peripheral ischemia.

### Surveillance Protocol

The follow-up of the patients was standardized including full medical history, clinical examination, and measurement of the ABI after month 1, 3, 6, 12, and yearly thereafter. Duplex ultrasound was performed if there was anamnestic or clinical suspicion of restenosis such as claudication, or if *a* > 0.15 decrease in ABI was measured. A peak systolic ratio > 2.4 at duplex sonography was considered indicative of a restenosis. If CT or MR angiography has been performed for another reason, the absence of restenosis at the target lesion was considered to be equivalent to duplex ultrasound. Primary patency (PP) was defined as freedom from > 50% restenosis, and target lesion revascularization (TLR) was defined as any repeated procedure (endovascular or surgical) for a restenosis at the initially treated CFA lesion (+ 1 cm proximally or distally to include edge phenomena); TLR was performed in patients with new limiting claudication or in case of CLI.

Efforts were made to minimize the number of patients lost to follow-up, including active tracking of the patients by contacting the patient or the family doctor, or by searching on the digitalized archive of public hospitals in the Canton Ticino. The number and the cause of death were equally registered.

### Statistical Analysis

Statistical analysis was performed using SPSS 23 and Medcalc software. Categorical variables are presented as numbers and percentages, and continuous and normally distributed variables are reported as mean ± standard deviation (SD) and as medians and interquartile ranges. Cumulative analysis of endpoints was assessed using Kaplan–Meier curves. Normality of restenosis and survival distributions was tested using the Kolmogorov–Smirnov test. Patients lost to follow-up were defined as censored for survival analysis. When analyzing time to restenosis, dead patients were considered as censored at the time point when they were last examined. Patients at risk at specific intervals are shown below the Kaplan–Meier curves, and standard errors (SEs) of cumulative event rates are provided. Two-tailed < .05 *p* value was used to set statistical significance.

## Results

This study includes 79 patients (age 70 ± 8.6 years; 68% men) with a median follow-up of 53 months (25–75% interquartile range, 24–86 months). All patients suffered from severe claudication (84%) or critical limb ischemia (Rutherford stage 4 and 5 in 15 limbs). Ninety-four limbs were treated with SAA; 69 lesions were limited to the CFA, 18 extended into the SFA, 6 into the deep femoral artery (DFA), and 1 into both the SFA and the DFA, respectively. Primary technical success was obtained in 93/94 limbs (99%; impossibility to cross the aortic bifurcation in 1 patient), and an outpatient procedure was possible in 92 procedures (98%). Complications occurred in 5 interventions (5.3%): 2 major puncture site hematomas, 1 arteriovenous fistula, 1 patient with distal cholesterol embolism, and 1 dissection of the access site artery, which had to be stented (all CIRSE classification grade 2; [12]). There was no death or major adverse event in the first 30 days. The ankle–brachial index (ABI) rose significantly from 0.71 ± 0.17 to 1.0 ± 0.2 (*p* < .001).

*Follow-up* During the structured follow-up period according to the surveillance protocol, 23 limbs (25%) showed a restenosis; of these, 15 (65%) were again successfully treated by angioplasty, 3 (13%) by surgery, and 5 (22%) conservatively, respectively. Restenosis did not depend on the length of the stents, nor was it more common in limbs with a compromised runoff. One patient (1.1%) underwent a major amputation of his leg due to extensive involvement of crural arteries. The ABI remained significantly higher as compared to the baseline value with 1.03 ± 0.2 after 1 year and 0.96 ± 0.21 at the last follow-up visit (*p* < .001 in all comparisons with pre-interventional ABI). The primary patency rate after 1, 3, 5, and 8 years was 96, 90, 78, and 63%, respectively. Five patients (6.3%) were lost to follow-up.

Accessibility for other interventions was possible either by puncturing the vessel just above or below the stent, or in single cases (*n* = 3) by inserting a sheath (up to a size of 6F) through the struts of the stent antegradely (*n* = 2) or retrogradely, without damaging its integrity.

**Fig. 1:**
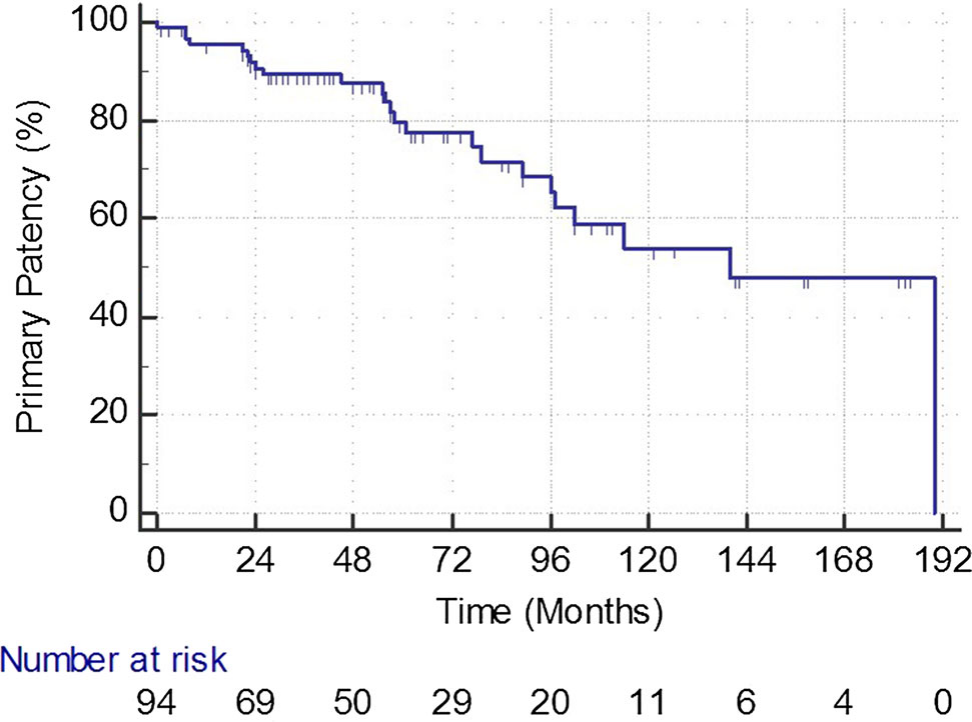
Primary patency rate during follow-up in 94 treated limbs. At each time point, SE is < 10%

**Fig. 2:**
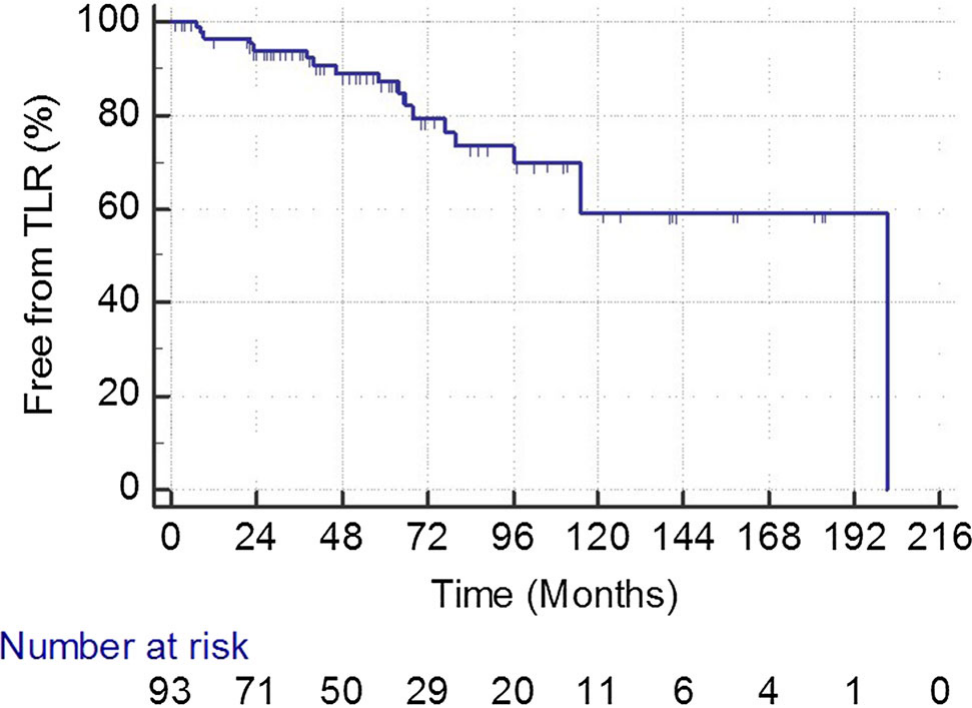
Target lesion revascularization (TLR) rate during follow-up in 93 successfully treated limbs. At each time point, SE is < 10%

**Fig. 3:**
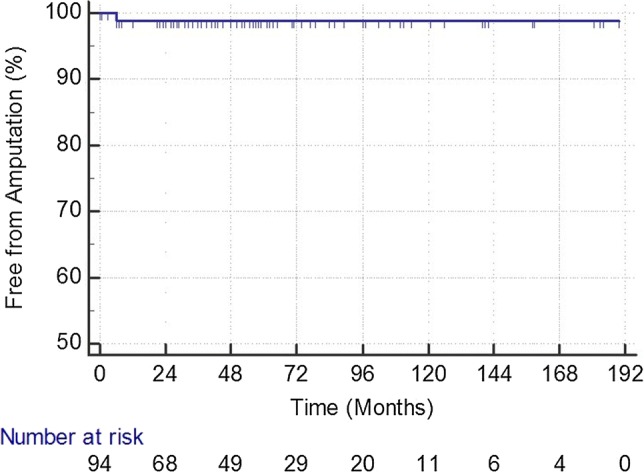
Amputation rate during follow-up in 94 treated limbs. At each time point, SE is < 10%

**Fig. 4:**
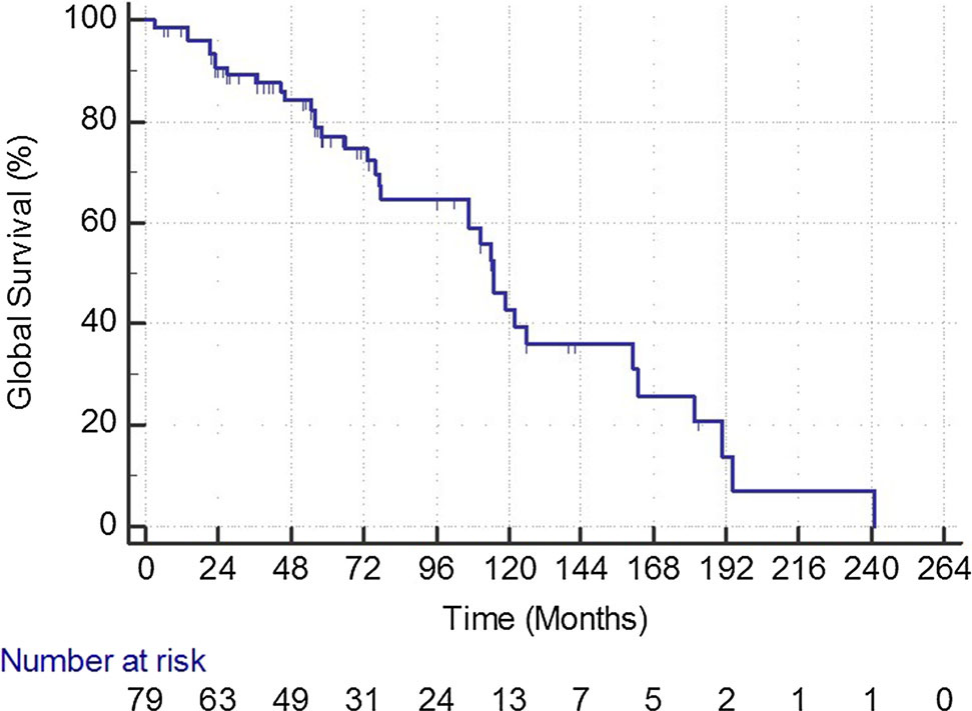
Global survival in 79 patients during follow-up. At each time point, SE is < 10%

During follow-up, 35 patients died (44.3%). The cause of death could be assessed in 31/35 patients. Twelve patients died of cardiovascular diseases, 6 had cancer, 4 sepsis, 3 chronic broncho-obstructive disease, 2 dementia, 2 cerebral hemorrhage, 1 liver cirrhosis, and 1 uremia, respectively (Figs. 1, 2, 3, and 4).

## Discussion

Obstructive lesions in the CFA are prone to restenosis: Baumann et al. in their series of 104 interventions in this area, with an initial technical success rate of 98%, found restenosis in 40% of the treated lesions after 1 year; only 27% of their patients had provisional stenting [7]. Bonvini et al. identified the use of stents as the only independent protective factor against procedural failure, 1-year restenosis, and target lesion revascularization (TLR); in their study, stenting was used in 37% of cases [8]. In a retrospective study with CFA lesions treated exclusively by balloon angioplasty, a relatively high re-intervention rate of 43% was found after 3 years in a group of patients with an elevated percentage of CLI [13]. A pooled analysis of common femoral vascular interventions showed a significantly higher mean primary patency at 12 months for routine stenting compared to a selective stenting strategy (91.4% vs. 75%) [11].

This study distinguishes itself from previous ones by the high number of included interventions, the strict adherence to an intervention protocol where every lesion was set to be stented exclusively by self-expanding stents, and ultimately the long follow-up period.

In 94 treated limbs, we found a high initial success rate of 99%, an overall restenosis rate of 25% and a peri-procedural complication rate of 5.3%; 98% of the interventions were done on an outpatient basis. Although CLI was present in 16% of the extremities, major amputation rate was low (1%). Restenosis were mostly treated by a second angioplasty. Remarkably, ABI values remained significantly higher during follow-up compared to baseline.

A similar retrospective study published recently included 36 patients for a mean follow-up of 64 months, with a primary patency rate after 3 and 5 years of 76 and 72%, respectively, a perioperative complication rate of 5%, and a cumulative mortality of 50%, which is in line with our results. Whereas our patients were mostly treated on an outpatient basis, those patients were hospitalized between 1 and 15 days [14].

Due to the study design, only indirect comparison is possible with patients treated by surgery. While immediate results by CFE compare well with endovascular procedures, the open approach has a better long-term patency rate with a PP up to 96% [5, 15], but local complications as well as systemic risks in patients treated by CFE are an issue [2].

Studies comparing SAA with CFE are rare. In the recently published French TECCO trial, patients with CFA lesions were randomized to undergo surgery (*n* = 61) or stenting (*n* = 56). The primary outcome was the morbidity and mortality rate within 30 days. Compared to the surgical group, the results were significantly in favor of the stenting group with a primary outcome in 26 and 6%, respectively. After a median follow-up of 2 years, the PP was similar in the two groups. The mean duration of hospitalization was significantly lower in the endovascular group. At 2 years, the primary sustained clinical improvement rate in the stenting group was 74%, which is slightly inferior to our 90% [16]. Interestingly, in that study one-third of the stents were balloon expandable, which in our experience is linked to a major tendency of restenosis.

A relevant finding in our study is the high mortality rate with 44.3% of the patients dying during follow-up. It seems that on the long run restenosis is not the main issue, which lessens the importance of this parameter when confronting endovascular and surgical procedures in the therapy of CFA lesions.

*Study Limitations* The main limitation of our study is the lack of a surgical control group, which allows only indirect comparison with what is still felt to be the gold standard for treating patients with CFA stenosis. Furthermore, our data were collected retrospectively, but based on a prospective protocol, which allowed us to keep the number of missing data low with only 5 (6.3%) patients lost to follow-up. Finally, the rate of stent fracture is not known as no plain film radiography was performed during follow-up.

## Conclusion

SAA of the CFA resulted in high initial success at the cost of a low peri-procedural complication rate and a moderate restenosis rate on follow-up. Economically, it may compare favorably to surgery with the majority of interventions feasible on an outpatient base. On follow-up, mortality rate almost doubled the risk of restenosis.
